# Intravascular donor monocytes play a central role in lung transplant ischaemia-reperfusion injury

**DOI:** 10.1136/thoraxjnl-2016-208977

**Published:** 2017-04-07

**Authors:** Kate Colette Tatham, Kieran Patrick O'Dea, Rosalba Romano, Hannah Elizabeth Donaldson, Kenji Wakabayashi, Brijesh Vipin Patel, Louit Thakuria, Andre Rudiger Simon, Padmini Sarathchandra, Harefield POPSTAR investigators, Nandor Marczin, Masao Takata

**Affiliations:** 1 Section of Anaesthetics, Pain Medicine and Intensive Care, Faculty of Medicine, Imperial College London, Chelsea and Westminster Hospital, London, UK; 2 Departments of Anaesthesia and Cardiothoracic Transplantation, Harefield Hospital, Royal Brompton and Harefield NHS Foundation Trust, Harefield, Middlesex, UK; 3 Faculty of Medicine, National Heart & Lung Institute, Imperial College, Heart Science Centre, Harefield Hospital, Harefield, Middlesex, UK

**Keywords:** Lung Transplantation, Innate Immunity, Macrophage Biology

## Abstract

**Rationale:**

Primary graft dysfunction in lung transplant recipients derives from the initial, largely leukocyte-dependent, ischaemia-reperfusion injury. Intravascular lung-marginated monocytes have been shown to play key roles in experimental acute lung injury, but their contribution to lung ischaemia-reperfusion injury post transplantation is unknown.

**Objective:**

To define the role of donor intravascular monocytes in lung transplant-related acute lung injury and primary graft dysfunction.

**Methods:**

Isolated perfused C57BL/6 murine lungs were subjected to warm ischaemia (2 hours) and reperfusion (2 hours) under normoxic conditions. Monocyte retention, activation phenotype and the effects of their depletion by intravenous clodronate-liposome treatment on lung inflammation and injury were determined. In human donor lung transplant samples, the presence and activation phenotype of monocytic cells (low side scatter, 27E10+, CD14+, HLA-DR+, CCR2+) were evaluated by flow cytometry and compared with post-implantation lung function.

**Results:**

In mouse lungs following ischaemia-reperfusion, substantial numbers of lung-marginated monocytes remained within the pulmonary microvasculature, with reduced L-selectin and increased CD86 expression indicating their activation. Monocyte depletion resulted in reductions in lung wet:dry ratios, bronchoalveolar lavage fluid protein, and perfusate levels of RAGE, MIP-2 and KC, while monocyte repletion resulted in a partial restoration of the injury. In human lungs, correlations were observed between pre-implantation donor monocyte numbers/their CD86 and TREM-1 expression and post-implantation lung dysfunction at 48 and 72 hours.

**Conclusions:**

These results indicate that lung-marginated intravascular monocytes are retained as a ‘passenger’ leukocyte population during lung transplantation, and play a key role in the development of transplant-associated ischaemia-reperfusion injury.

**Key messages**

**What is the key question?**

- Resident lung mononuclear phagocytes have been implicated in ischaemia-reperfusion injury and the development of primary graft dysfunction following lung transplantation, but the contribution of lung intravascular monocytes to lung transplant-related injury is not known.

**What is the bottom line?**

- Retention and activation of intravascular monocytes in perfused murine and human donor lungs suggests an important and previously unrecognised role as passenger leukocytes contributing to lung injury and primary graft dysfunction, emphasising their potential as therapeutic targets in lung transplantation.

**Why read on?**

- This study suggests that vascular monocytes play a key role in lung injury following ischaemia-reperfusion.

## Introduction

Lung transplantation is the final treatment option for patients with end-stage lung disease. Primary graft dysfunction (PGD) plays a major role in recipient morbidity and mortality in the days following transplantation, and negatively impacts on long-term graft survival.^1^ Ischaemia-reperfusion (I/R) injury is an inevitable feature of the lung transplantation process and the primary pathophysiological mechanism responsible for PGD.^2^ ^3^ While efforts to manage the ischaemic insult have improved graft success, effective therapies to combat the mechanisms of I/R injury, and hence reduce PGD, remain elusive.

Inflammatory leukocytes (mononuclear phagocytes and neutrophils) play a central role in I/R-induced acute lung injury pathogenesis.^4^ ^5^ Animal model studies of lung I/R provide strong evidence that the injury development is bimodal,^5^ ^6^ with an early response that is considered to be largely dependent on alveolar macrophages but independent of neutrophils, and a delayed secondary injury phase, involving recruitment of circulating neutrophils to the inflamed pulmonary vasculature. In human lung transplantation, higher levels of the neutrophil chemoattractant IL-8 in donor lung tissue or bronchoalveolar lavage (BAL) fluid have been linked to PGD severity, consistent with a two-step process involving neutrophils as late stage effector cells.^7^ ^8^ Thus, the early resident leukocyte responses are likely to play a significant role in ‘initiating’ I/R lung injury during transplantation, representing potentially important therapeutic targets for reducing PGD.

In addition to alveolar macrophages, other substantial mononuclear phagocytic populations are indeed present within the interstitial and vascular compartments of the donor lungs.^9^ Monocytes marginated in the narrow pulmonary capillaries have been shown to play a crucial role in enhancing acute lung injury resulting from microbial or mechanical insults in preclinical models.^10–12^ There is also evidence from human transplant studies that donor lung intravascular monocytes are not completely removed by perfusion and carried over as ‘passenger’ cells, with their presence shown in post-perfusion lung transplant tissue^13^ as well as in the circulation of recipients.^13^ ^14^ However, the potential contribution of these intravascular passenger monocytes to the pathogenesis of I/R injury, and thus PGD, has not been studied.

We hypothesised that donor lung-marginated, intravascular monocytes, exposed directly to the I/R-induced vascular stress and in close contact with the pulmonary capillary endothelium, would play a key role in development of transplant-related I/R lung injury, and thus PGD. We investigated this hypothesis using a mouse isolated perfused lung (IPL) model of transplant-related early I/R injury, combined with intravascular monocyte depletion and repletion treatments. We then evaluated numbers and phenotypes of mononuclear phagocytes within pre-implantation lungs and studied their relationships with post-implantation gas exchange and PGD severity in human lung transplantation.

## Methods

Detailed methods are provided in the online supplementary methods.

10.1136/thoraxjnl-2016-208977.supp1 Supplementary data

### Animal experimentation

All protocols were approved by the Imperial College Ethical Review Board and UK Home Office in accordance with the Animals (Scientific Procedures) Act 1986, UK, and performed in line with the ARRIVE guidelines (see online supplementary methods). Male C57BL/6 mice aged 8–12 weeks (24–28 g) were used.

### Isolated perfused lung

As previously described,^11^ anaesthetised mice were mounted on an IPL system, and after exsanguination and thoracotomy, their pulmonary artery and left atrium cannulated. Lung perfusion with supplemented RPMI-1640 and ventilation was commenced (see online supplementary figure E1). After an initial washout period, lungs underwent either 15 min of open perfusion (compartment protocol, see online supplementary figure E2A), 2 hours of ischaemia/2 hours of reperfusion (I/R protocol, see online supplementary figure E2B) or adoptive transfer of blood monocytes prior to I/R (adoptive transfer protocol, see online supplementary figure E2C).

For monocyte depletion experiments, mice received intraperitoneal clodronate liposomes (Formumax Scientific, Palo Alto, California, USA) 24 hours prior to the I/R protocol. For adoptive transfer experiments, monocytes were isolated from blood of donor mice using a negative magnetic bead selection kit (StemCell Technologies, Grenoble, France) and infused into the IPL.

### Sample analysis

On completion of the protocols, left lungs were tied off for wet:dry weight ratio determination, and remaining lung lobes underwent BAL and disaggregation for flow cytometric analysis. BAL fluid was analysed for protein content, and perfusate soluble inflammatory markers (MIP-2, KC, MCP-1, RAGE, TNF-α, IL-β, IL-6) were quantified by ELISA. Lung cell suspensions were prepared by tissue disaggregation in fixative in a gentleMACS dissociator (Miltenyi, Bisley, UK) and samples were passed through a 40 µm strainer and washed.^15^ ^16^ Cell samples were incubated with the antibodies in online supplementary table E1.

### Human samples

All patients provided informed consent to participate in the ethically approved POPSTAR study (Peri-OPerative Study of lung Transplantation and Acute Respiratory failure; reference 13/LO/0152), investigating surgical and technological aspects of transplantation. Human samples and patient data were stored in accordance with the study protocol at the Royal Brompton and Harefield NHS Foundation Trust, UK. Lower lobe biopsies (∼3 cm×1 cm×1 cm) were obtained from a total of 13 donor lungs (already flushed at the time of retrieval), immediately prior to implantation, and stored on ice. Lung tissue was minced and disaggregated with Liberase/DNAse for 20 min at 37°C, passed through a 40 µm strainer and washed. Lung cell suspensions and blood samples from healthy volunteers were incubated with the antibodies in online supplementary table E3. Electron micrographs were prepared for, and acquired by, the Transmission Electronic Microscope JEOL 1200EX, detailed in the online supplementary methods.

### Flow cytometry

Antibody-stained cell samples were analysed using a Cyan flow cytometer (Beckton Coulter, High Wycombe, UK) with FlowJo software (V.10.0.8, Ashland, Oregon, USA).

### Statistics

Normality was determined using QQ plots and Shapiro-Wilk tests. Group comparisons were made by Student's t tests or Mann–Whitney U tests, or by ANOVA with Bonferroni tests or Kruskal–Wallis with Dunn's tests for more than two groups. Correlation analysis was performed with Spearman rank test. Data are presented as mean±SD (parametric) or median±IQR (non-parametric). Statistical significance was defined as p<0.05.

## Results

### Pulmonary intravascular monocytes are retained during lung perfusion

**Figure 1:**
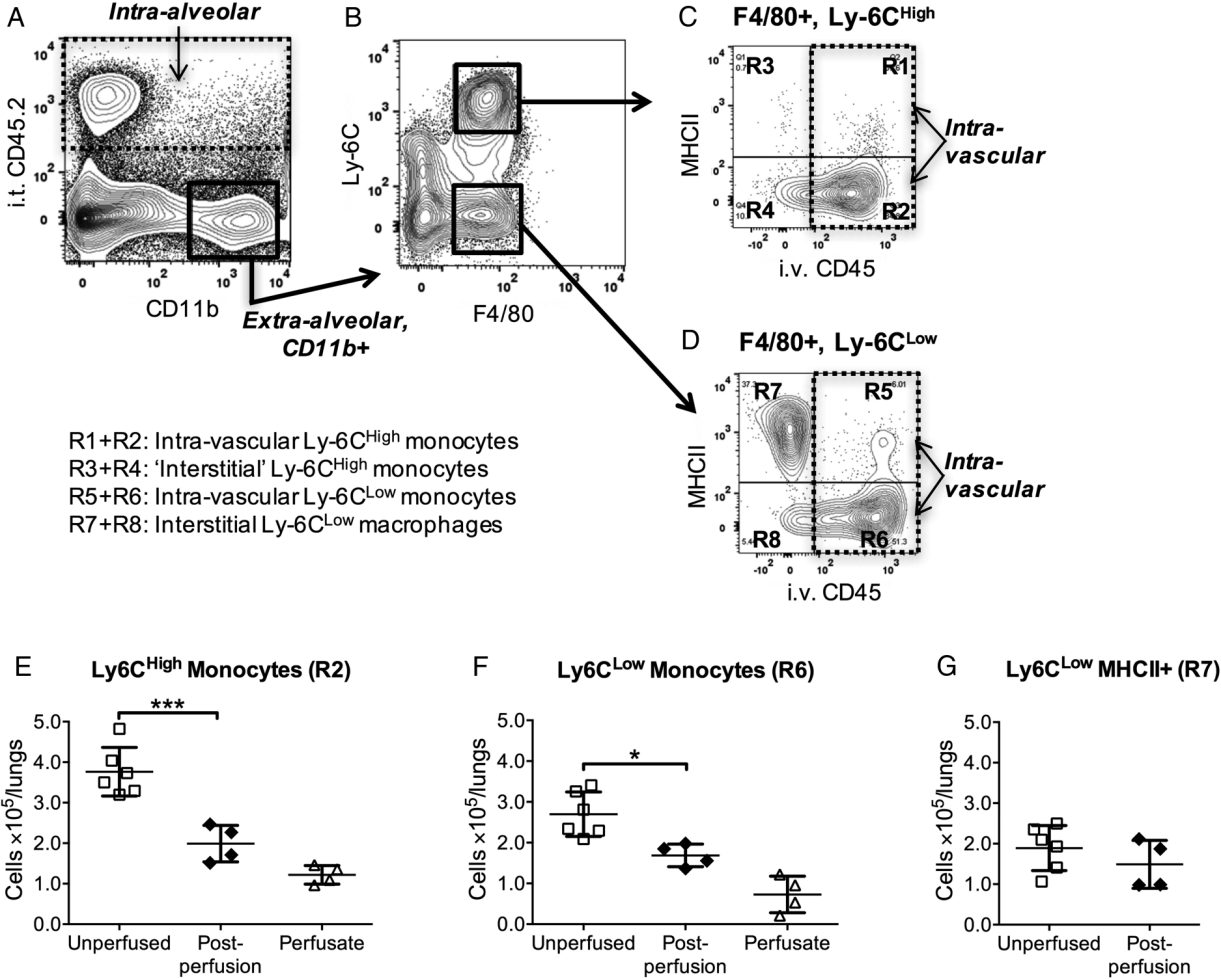
Pulmonary intravascular lung monocytes are retained during lung perfusion. The location and phenotype of monocyte subsets in the lungs were determined by dual compartment, intra-vascular and intra-alveolar leukocyte staining. Mice were injected intravenously (i.v.) with anti-CD45 (PE-CF594) before anaesthetic overdose, followed by intra-tracheal (i.t.) instillation of anti-CD45.2 (APC). To analyse mononuclear leukocytes within the lung cell suspensions, extra-alveolar leukocytes were first identified as CD45.2− and CD11b+ cells (A). In these cell populations, monocytes/macrophages were further identified as F4/80+ and subdivided into Ly6C^High^ and ^Low^ subsets (B). Intravascular monocytes were identified as anti-CD45 PE-CF594+ (C and D). MHCII was used as a marker for interstitial CD11b+, F4/80+ macrophages. To evaluate intravascular monocyte removal during perfusion, lungs were flushed with open circuit perfusion for 15 min after compartmental antibody staining. Only partial reduction in intravascular monocyte numbers was observed following washout (black diamonds) compared with non-surgical controls (white boxes) with both intravascular Ly6C^High^ (E) and Ly6C^Low^ (F) populations. Numbers of interstitial Ly6C^Low^, MHCII+ macrophages (G) were not significantly changed, confirming their extravascular location. These numbers of intravascular monocyte subsets far exceeded those expected in an unlikely situation when residual blood within the pulmonary vasculature (estimated ∼50 µL as maximum) was not washed out, and totally preserved within the lung despite this extended perfusion, and hence can be ascribed to a marginated pool. Data are displayed as mean±SD, and analysed by t test. n=4–6, *p<0.05, ***p<0.001.

To investigate the potential for retention of intravascular monocytes within the lungs during ex vivo perfusion, we first determined the size of the lung-marginated monocyte pool (figure 1), using flow cytometry and a dual-compartment (vascular and alveolar) antibody staining technique developed in our laboratory.^16^ Intra-vascular leukocytes were identified by intravenous injection of mice with an anti-CD45 antibody (Clone 30-F11, PE-CF594) and intra-alveolar leukocytes by intra-tracheal instillation of anti-CD45.2 (Clone 104, APC). Cells double-negative for intravenous CD45 and intra-tracheal CD45.2 included interstitial leukocytes, while double-positive cells were rarely observed (<1%), indicating compartment specificity of the method. Intravascular Ly6C^High^ and Ly6C^Low^ monocytes were clearly identified using their cell surface marker characteristics (CD11b+, F4/80+, MHCII−) combined with this compartmental staining technique. The lung Ly6C^High^ monocytes were largely situated in the intravascular space (figure 1C:R2), with a small interstitial subpopulation (figure 1C:R4) that may be intravascular but not fully stained by injected intravenous antibody. Similarly, the lung Ly6C^Low^ monocytes were largely intravascular (figure 1D:R6). There was a population of mononuclear phagocytes in the interstitial space, which was CD11b+, F4/80+, Ly6C^Low^ but MHCII+ (figure 1D:R7), and their number was comparable to that of intravascular Ly6C^Low^ monocytes. These cells are equivalent to the interstitial macrophage and/or dendritic cell populations previously described by others^17^ and ourselves.^16^ The alveolar compartment was comprised predominantly (>99%) of alveolar macrophages (CD11b−, F4/80^High^, CD11C^High^).

Monocyte retention during extended ex vivo perfusion was then determined following dual compartment labelling (figure 1E–G). Following open-circuit non-recirculating perfusion (RPMI-1640, human albumin 4%, 25 mL/kg/min) for 15 min, numbers of intravascular Ly6C^High^ monocytes were reduced to only approximately one-half (54.1±12.3%) compared with control non-perfused lungs, and an even larger proportion (62.5±10.3%) of Ly6C^Low^ monocytes were retained. As expected, numbers of ‘interstitial’ (Ly6C^Low^, CD11b+, F4/80+, MHCII+) cells were not altered significantly by this perfusion procedure. The total number of monocytes (Ly6C^High^+Ly6C^Low^) collected in the effluent from the perfused lungs (‘perfusate’) approximated to the difference between the (non-perfused) controls and remaining (retained) monocytes in the perfused lungs.

### Activation of intravascular monocytes during lung I/R

In situ mouse IPLs were subjected to a protocol of warm normoxic ischaemia (2 hours) and reperfusion (2 hours in a recirculating manner), incorporating three 5 min open-circuit washout periods pre ischaemia, post ischaemia and post reperfusion (see online supplementary figure E2B). Intravascular monocytes were identified as Ly6C^High^ or Ly6C^Low^, CD11b+, F4/80+ and MHCII− cells in these experiments, based on the compartmental analysis of lung leukocytes in figure 1. Substantial proportions of the intravascular Ly6C^High^ and Ly6C^Low^ monocyte populations were retained at the end of I/R, comparable to, or somewhat higher than, the 15 min washout (Ly6C^High^ 79.8±20.7%; Ly6C^Low^ 69.7±19.3%), presumably due to I/R-induced cell activation. Such retention of intravascular monocytes, exposed to the ischaemic insult and present subsequently during the reperfusion period, could play a significant role in the progression of lung injury in this ex vivo I/R model.

**Figure 2:**
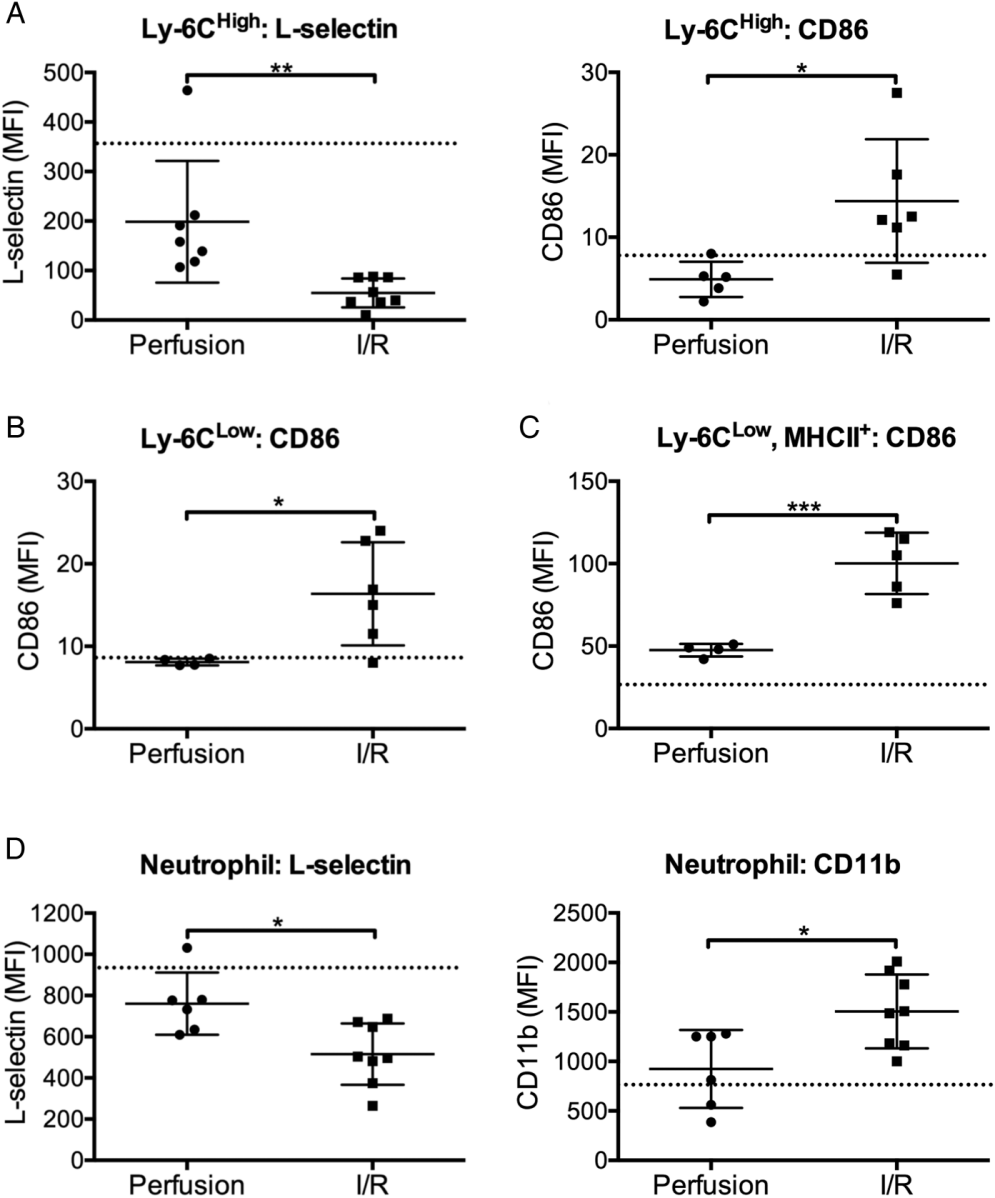
Activation of retained lung monocytes and neutrophils during ischaemia-reperfusion (I/R). Monocyte and neutrophil activation during I/R was indicated by changes in expression of their respective surface activation markers relative to 2 hours of perfusion only, with values for those from untreated (non-surgical) control mice indicated by a dotted line. Ly6C^High^ monocyte activation was indicated by L-selectin shedding and CD86 upregulation (A); Ly6C^Low^ monocytes and interstitial macrophages by increased CD86 expression (B and C); and neutrophils by L-selectin shedding and increased surface CD11b expression (D). Data are displayed as mean±SD and analysed by t tests. n=4–8, *p<0.05, **p<0.01, ***p<0.001.

To evaluate the role of these retained intravascular monocytes in the development of I/R injury, we first determined whether they became activated during the IPL-I/R procedure (figure 2A–D). On Ly6C^High^ monocytes, L-selectin expression, a classical ‘endpoint’ marker of non-transcriptional cell activation,^18^ was substantially reduced, while CD86, an inflammation-induced T-lymphocyte costimulatory molecule,^19^ was upregulated at the end of the I/R protocol. On intra-vascular Ly6C^Low^/MHCII− monocytes, which do not classically express appreciable amounts of L-selectin, I/R-induced activation was indicated by CD86 upregulation. This response was also observed on the interstitial MHCII+/Ly6C^Low^ macrophage population, indicating cross-compartmental activation as a result of I/R. I/R-dependent neutrophil activation was also observed with reduction in L-selectin expression and increased cell surface CD11b expression.

### Depletion of monocytes attenuates I/R-induced lung injury and inflammation

To further determine the role of pulmonary intravascular monocytes in I/R-induced lung injury, we pretreated mice by intravenous injection with clodronate liposomes to produce intravascular compartment-specific monocyte depletion.^20^ This treatment was effective in significantly reducing the lung intravascular monocyte population whilst leaving the neutrophils, interstitial (CD11b+, F4/80+, MHCII+) and alveolar macrophage populations intact (table 1).

**Table 1:** Lung leukocyte numbers 24 hours after clodronate-liposome injection

	Vascular Ly6C^High^ monocytes ×10^5^/lungs	Vascular Ly6C^Low^ monocytes ×10^5^/lungs	Interstitial MHCII+ macrophages ×10^5^/lungs	Alveolar macrophages ×10^5^/lungs	Vascular neutrophils ×10^5^/lungs
Control mice	4.51 (3.3–7.3)	3.15 (3.0–4.8)	0.90 (0.7–1.3)	18.8 (17.0–24.4)	5.42 (4.1–7.8)
Clodronate treated mice	1.98 (1.6–2.3)***	0.72 (0.3–1.0)**	0.70 (0.5–1.6)	18.98 (18.5–21.3)	10.0 (6.3–18.7)

Lung leukocyte numbers were quantified via flow cytometry in control (non-surgical) mice, with and without prior clodronate administration. Data were analysed by Mann–Whitney U test (median±IQR×10^5^/lungs); n=9 and 6, **p<0.01, ***p<0.001 vs control mice.

**Figure 3:**
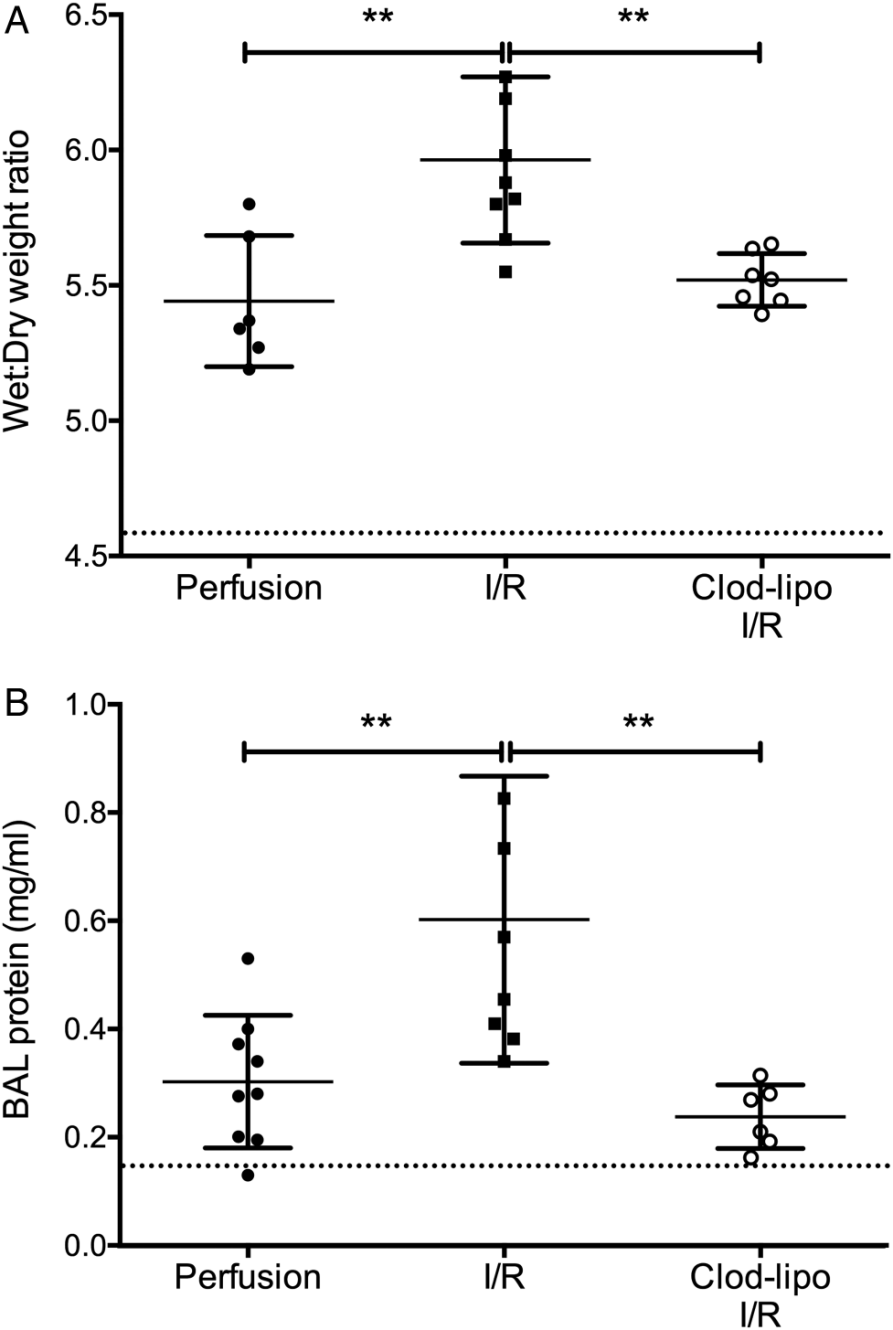
Depletion of intravascular monocytes attenuates ischaemia-reperfusion (I/R)-induced lung injury. I/R-induced lung injury was indicated by increases in lung wet:dry weight ratios (A) and bronchoalveolar lavage (BAL) fluid protein production (B). BAL fluid protein and wet:dry ratios were increased with 2 hours of perfusion of the lungs alone, but they were not significantly different from untreated non-surgical controls (dotted line). Clodronate-liposome (clod-lipo) depletion of intravascular monocytes restored these indices to baseline. Data are displayed as mean±SD and analysed by one-way ANOVA with Bonferroni correction tests. n=6–9, **p<0.01.

I/R-induced lung injury, as determined by increased lung wet:dry weight ratio and BAL fluid protein levels, was substantially reduced in monocyte pre-depleted lungs, with both indices attenuated to the levels seen in perfusion-only controls (figure 3).

**Figure 4:**
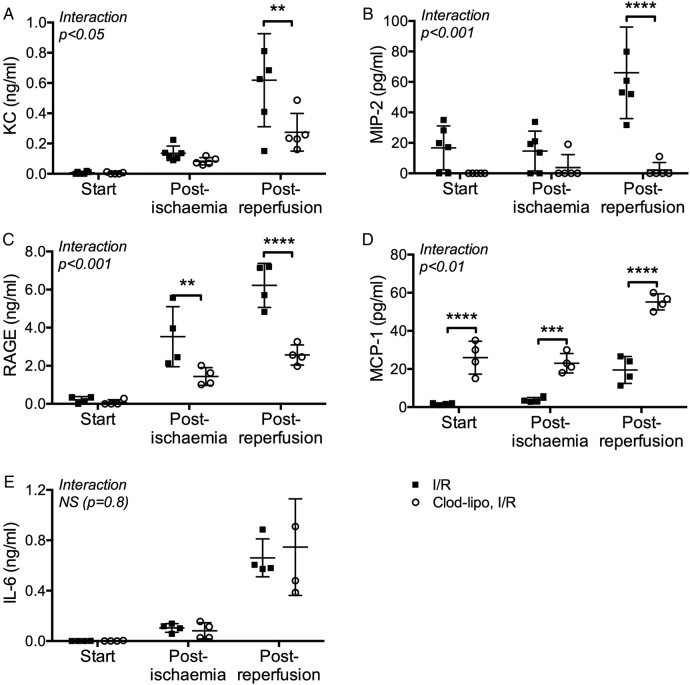
Intravascular monocyte depletion modifies soluble mediator release during lung ischaemia-reperfusion (I/R). Lungs from normal (black squares) or clodronate-liposome pretreated (white circles) mice were subjected to I/R, with perfusate samples obtained pre ischaemia, post ischaemia and post reperfusion. Levels of soluble KC (A), MIP-2 (B) RAGE (C), MCP-1 (D) and IL-6 (E) were determined by ELISA. Increases in KC, MIP-2 and RAGE post ischaemia and post reperfusion were reduced by clodronate-induced monocyte depletion, whereas this treatment resulted in higher MCP-1 levels at all sampling points. Data are displayed as mean±SD, and analysed by two-way ANOVA with t tests with Bonferroni correction. n=4–6, **p<0.01, ***p<0.001, ****p<0.0001.

Lung perfusates sampled pre and post ischaemia and at the end of the reperfusion period showed increasing levels of KC, MIP-2 and RAGE during the protocol (figure 4A–C). Depletion of monocytes by intravascular clodronate-liposome treatment produced a considerable reduction in the levels of these markers, which was clearly evident in the case of RAGE at the immediate post-ischaemia time point, indicating an early role for monocytes in development of I/R injury. However, MCP-1 levels were higher in clodronate-treated mouse lungs than normal lungs throughout the I/R protocol (figure 4D), consistent with reduced MCP-1 absorption by monocytes, as previously described,^21^ ^22^ and confirming monocyte-specific effects of the depletion method. TNF and IL-1β levels, which have previously been reported to be elevated in lung I/R models,^23^ ^24^ were negligible in the vascular compartment, while IL-6 was increased, but unaffected by monocyte depletion (figure 4E), indicating alternative cell sources or mechanism of induction. We also demonstrated a reduction in I/R-induced upregulation of CD11b on lung neutrophils in monocyte-depleted mice (I/R: 1506±373MFI; clodronate-liposome + I/R: 540±176MFI, p<0.0001), consistent with the observed lower release of neutrophil-activating chemokines KC and MIP-2.

### Adoptive transfer of blood-derived monocytes partially restores I/R-induced acute lung injury

**Figure 5:**
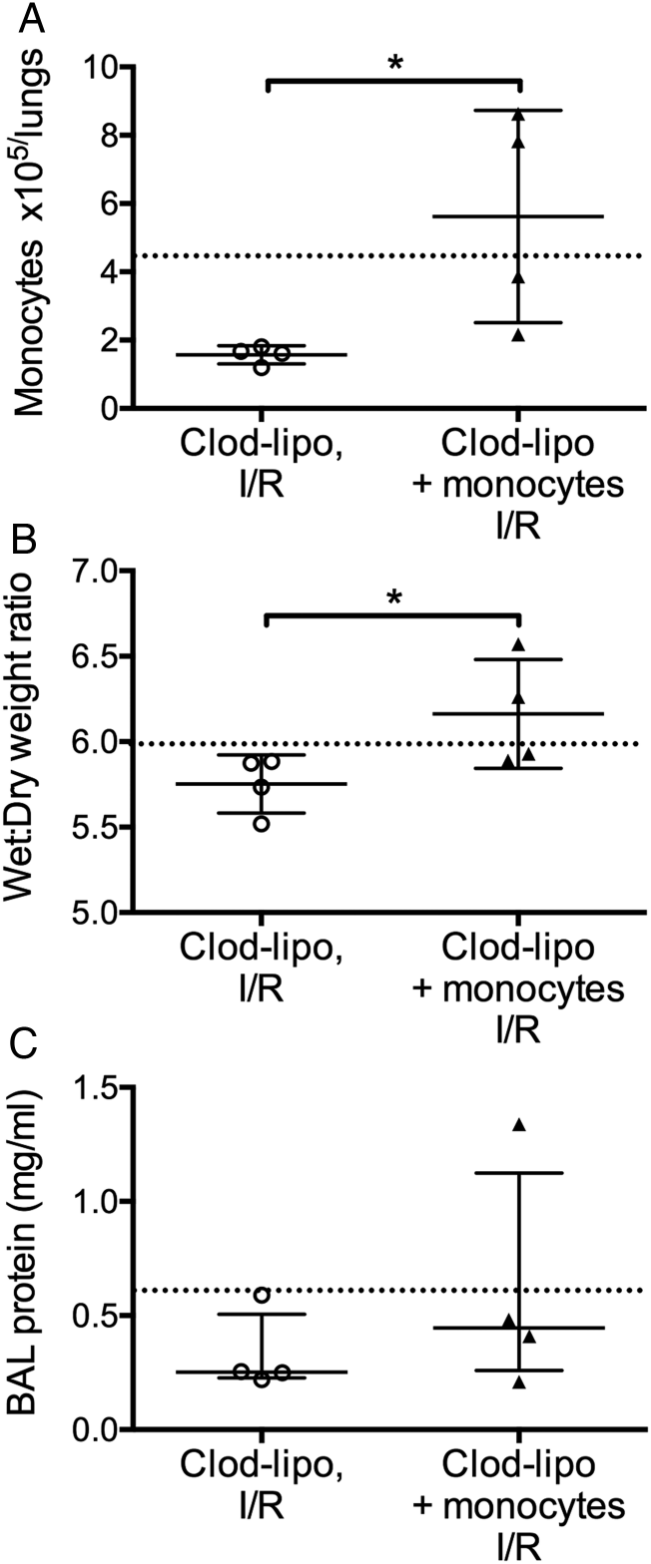
Adoptive transfer of monocytes restores ischaemia-reperfusion (I/R)-induced lung injury in monocyte-depleted lungs. Isolated lungs from monocyte-depleted mice were infused slowly (1 min) with normal blood-derived monocytes and recirculated for 10 min prior to initiation of the ischaemic period. At the end of I/R, Ly6C^High^ monocyte numbers were found to be elevated in infused lungs (A). Lung wet:dry weight ratios (B) and bronchoalveolar lavage (BAL) protein levels (C) were increased in monocyte-infused lungs, indicating monocyte-dependent IR injury. Post-I/R treatment values obtained in previous experiments in normal (non-monocyte-depleted) I/R mice are indicated by a dotted line. Data are analysed by t tests (A and B; mean±SD) or by Mann–Whitney U tests (C; median±IQR). n=4, *p<0.05. clod-lipo, clodronate-liposome.

To confirm the role of intravascular lung-marginated monocytes in I/R-induced injury response, monocytes from normal mice were adoptively transferred into isolated lungs of monocyte-depleted mice (figure 5). Monocytes were enriched by negative selection from the blood of donor mice and injected via the pulmonary artery into pre-flushed lungs prior to the ischaemic period. This method restored monocytes to the levels approaching those in normal I/R lungs seen in table 1, albeit with a larger variance that may reflect variable margination rates for ex vivo isolated monocytes and/or their potential loss within the perfusion circuit (eg, adherence to tubing). Both lung injury parameters were increased by adoptive transfer of monocytes, although this did not reach statistical significance in the case of BAL protein.

### Analysis of monocytes in pre-implantation human lungs

**Figure 6:**
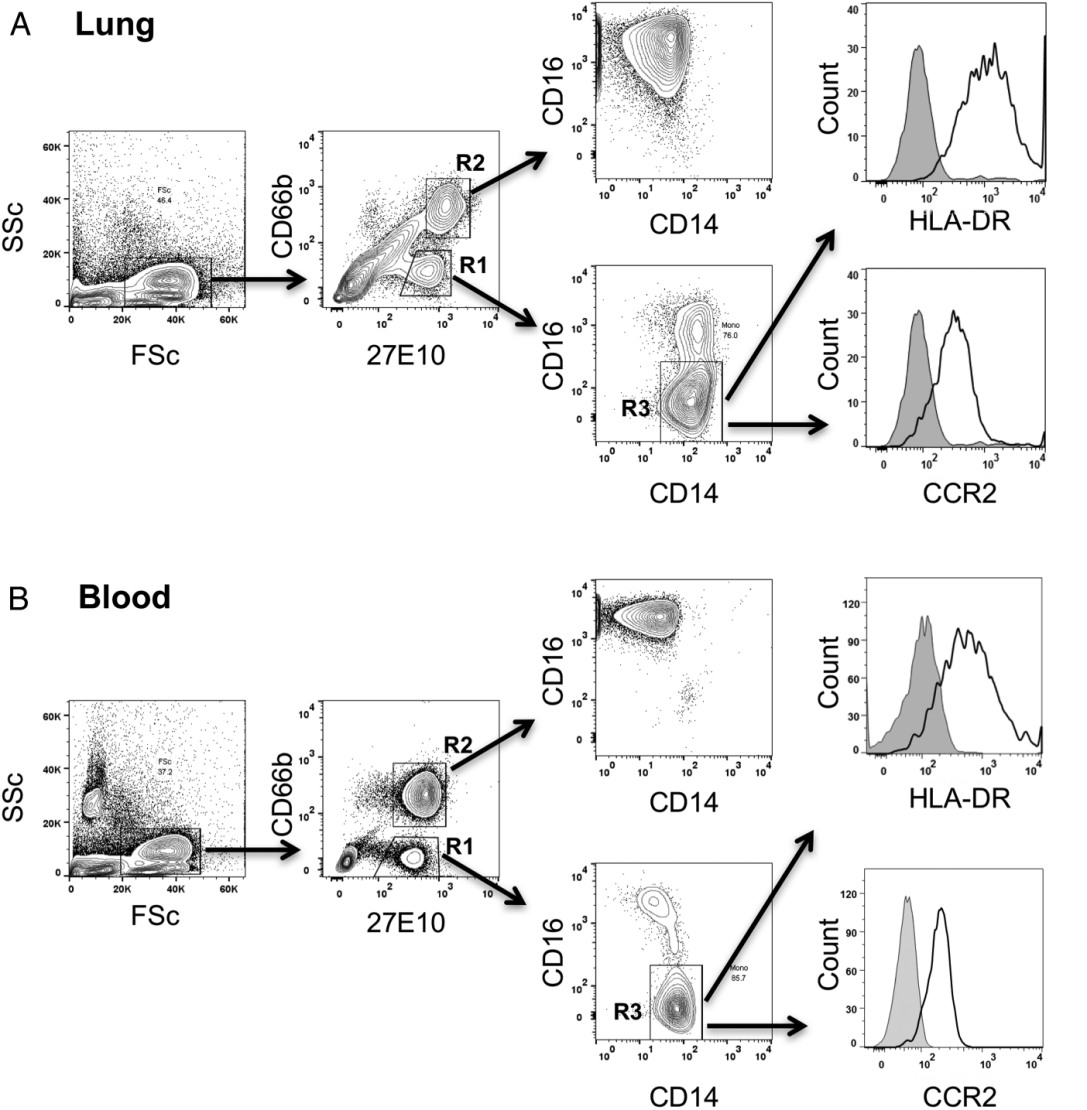
Identification of monocyte subsets in human donor lung grafts. Single cell suspensions prepared from donor lung tissue were antibody stained for surface markers, and then fixed and permeabilised for intracellular detection of the monocyte and neutrophil S100A8/A9 antigen using the 27E10 monoclonal antibody (A). Classical subset monocytes were identified in lungs as 27E10^High^, CD66b– (R1) and CD14+, CD16– (R3) events. Granulocytes were identified as 27E10^High^, CD66b+ events (R2). Using the same staining protocol, comparable staining characteristics were observed for monocytes in healthy volunteer whole blood (B), including similar expression levels of HLA-DR and CCR2.

To assess the relationship between donor monocytes sequestered within human transplant lungs and their potential contribution to development of PGD in recipients, a pilot study was performed in which a total of 13 lungs were analysed (see online supplementary table E2 for demographic, physiological and outcome data). Donor monocytes in lung cell suspensions, prepared from biopsy samples taken immediately before implantation, were identified as low side scatter/27E10+/CD14+ events, distinguished from 27E10+ granulocytes by absence of CD66b expression (figure 6). The flow cytometry profile of these lung-associated monocytes was identical to that of the CD14+/CD16− phenotype of classical subset monocytes in healthy volunteer blood, with further confirmation based on their comparable HLA-DR and CCR2 expression. We limited our analysis to these classical CD14+/CD16− subset monocytes in lungs, omitting CD14+/CD16+ cells due to potential overlap with lung macrophages expressing CD16.^25^

**Figure 7:**
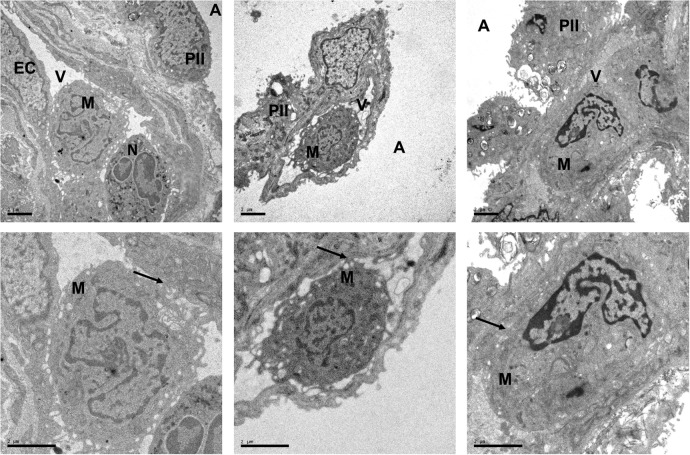
Electron micrographs showing intravascular monocyte in pre-implantation lung biopsies. Electron micrographs from three different lung allografts are shown. Samples were obtained at the end of the cold ischaemia, prior to implantation. Scale=2 μm. The endothelial cells (ECs) covering the vascular (V) side of the alveolar–capillary barrier appear unmodified, with preserved mitochondria. Moreover, no significant changes are exhibited by the monolayer of type I pneumocytes, lining the alveolar space (A), or the type II pneumocytes (PII). Despite the flushing at the time of the retrieval, monocytes (M), with typical horseshoe-like nuclei, and neutrophils (N), with bi-lobed nuclei, are seen within the pulmonary vasculature. These micrographs also demonstrate evidence for various grades of monocyte–endothelial interactions (arrows), including multiple connections with the ECs consistent with tethering and manifest adhesion.

Large numbers of donor monocytes (3.8±2.0×10^6^ cells/g dry weight) and granulocytes (8.1±6.7×10^6^ cells/g) were still found in tissue from these lungs before implantation, despite the fact that they had been subjected to the standard anterograde and retrograde (5L perfusate) perfusion protocols to remove blood elements prior to implantation. Consistent with this, H&E and immunohistochemistry microscopy showed significant presence of leukocytes in the pre-implantation lung biopsies (see online supplementary figure E3). Electron microscopy (EM) provided further clear evidence of monocyte margination within pulmonary capillaries, where they were found in close apposition with endothelial cells (figure 7).

**Figure 8:**
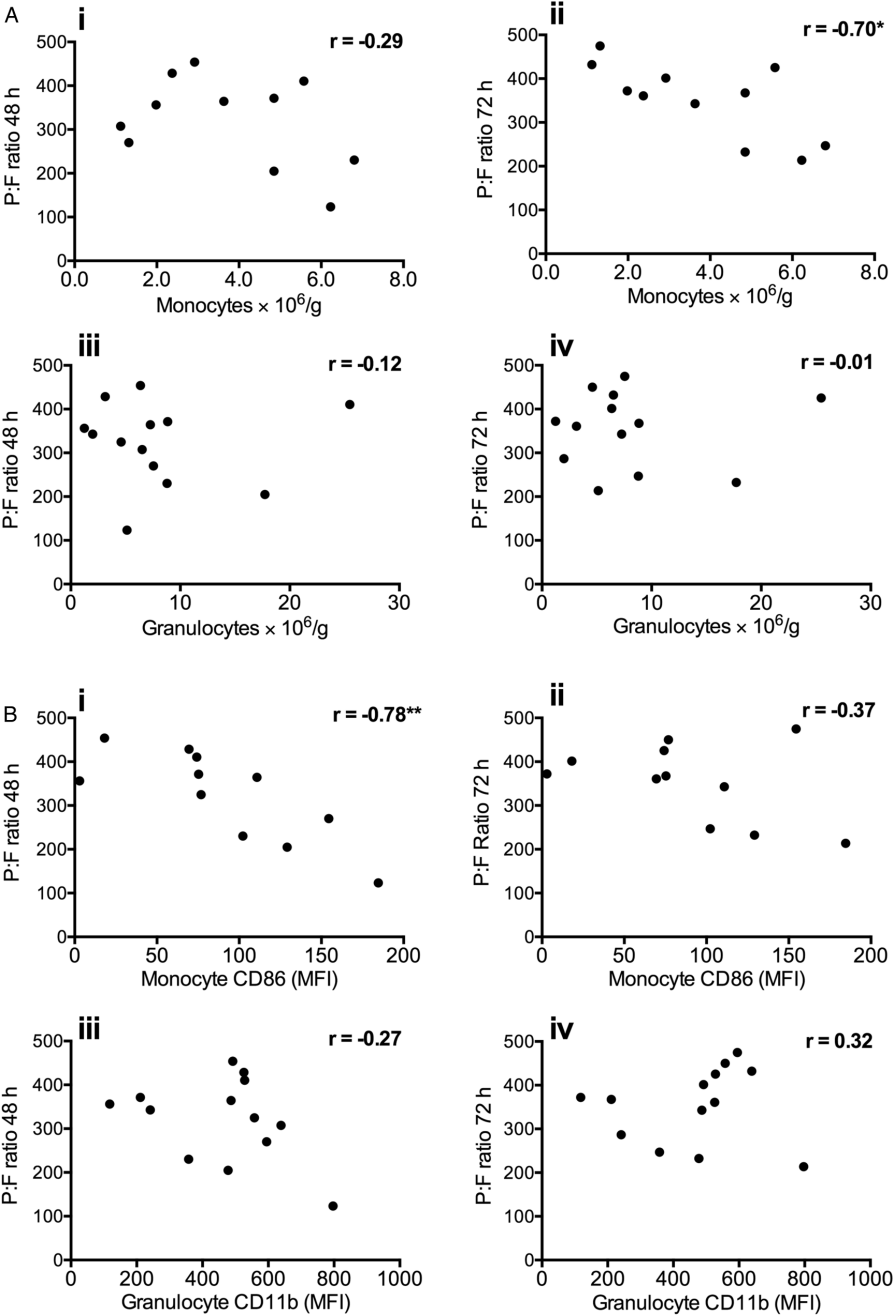
Correlation of donor lung monocyte numbers and activation state with P:F ratios following transplantation. Numbers of monocytes retained in donor lungs post harvest were found to negatively correlate with P:F ratios at 72 hours post implantation (p<0.5) (Aii). Donor lung granulocyte numbers did not correlate with P:F ratios at either 48 or 72 hours (A; iii–iv). Donor lung monocyte activation (CD86 expression) correlated negatively with P:F ratio at 48 hours (Bi). No correlation was seen with granulocyte activation (CD11b), at either time point (B; iii–iv). Data are analysed by Spearman's rank test (showing r values *p<0.05, **p<0.01), n=11–13.

**Figure 9:**
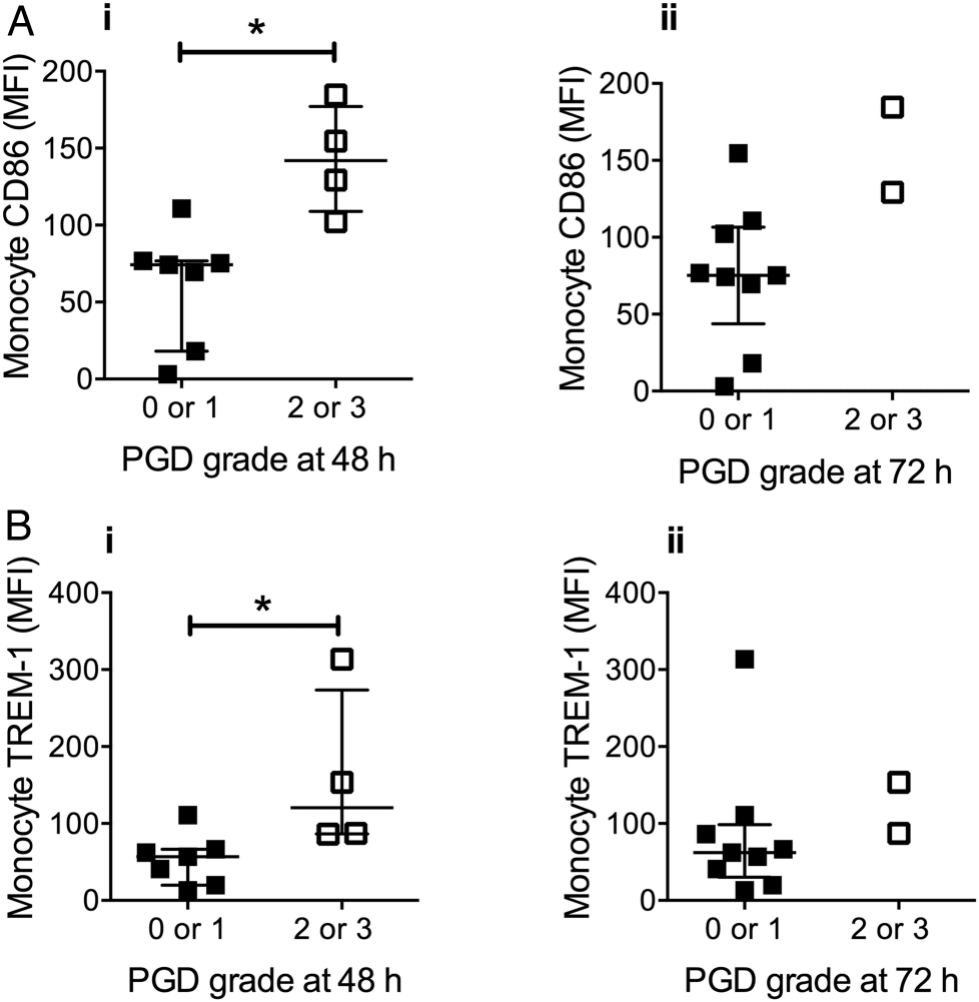
Donor monocyte activation is associated with primary graft dysfunction (PGD) severity. Expression of CD86 and TREM-1 on donor lung monocytes pre implantation was higher in lungs that developed grade 2 or 3 PGD at 48 hours post transplantation (Ai and Bi). Data are displayed as median±IQR and analysed by Mann–Whitney U tests. n=11, *p<0.05. (NB 2 samples were lost from the donor monocyte CD86/TREM-1 analysis due to technical difficulty).

Comparison of donor monocyte numbers with P:F ratios at 48 and 72 hours post implantation (figure 8A) revealed a high level of negative correlation at 72 hours (r=−0.70, p=0.016), although there was only a weak non-significant correlation at 48 hours. In contrast, there was no apparent relationship between donor granulocytes and P:F ratios at either time point. Examination of donor monocyte CD86 and TREM-1 expression also indicated a relationship between the CD86 levels and lung dysfunction, showing a high inverse correlation with P:F ratios at 48 hours (r=−0.78, p=0.004), while no clear relationship was found between neutrophil CD11b expression and P:F ratios (figure 8B). The significance of the donor monocyte activation status and severity of PGD was further assessed by analysis of CD86/TREM-1 expression and PGD scores at 48 and 72 hours (figure 9). CD86 expression was higher in transplant recipients with PGD grade III at 48 hours, and remained higher in those recipients at 72 hours. Levels of TREM-1 expression were also higher at 48 hours, but the inverse correlation with P:F levels did not reach significance (r=−0.57, p=0.078).

## Discussion

A role for pulmonary intravascular monocytes as passenger leukocytes, contributing to I/R injury and PGD following lung transplantation, is not widely recognised.^26^ We demonstrate, for the first time, that intravascular monocytes retained in an IPL model become activated and contribute significantly to the development of I/R lung injury. The clinical significance of these observations was supported by our pilot study with human donor lungs, where monocyte numbers and their expression of surface activation markers were associated with reduced postoperative gas exchange and development of PGD in recipients. As a substantial intravascular population with immediate exposure to and interactions with donor and recipient environments, donor-derived passenger monocytes could represent a novel therapeutic target to improve the quality and function of lung allografts.

We found that substantial numbers of intravascular monocytes were still present after prolonged non-recirculating perfusion in murine lungs. A similar degree of monocyte retention was found after lung I/R, where non-recirculating perfusion was repeated three times. Sequestration of leukocytes within the pulmonary microvasculature, in particular monocytes, during ex vivo perfusion of rodent lungs has previously been reported,^20^ ^27^ and the traditional method to define lung tissue (interstitial) leukocytes as cells remaining after lung perfusion is now recognised as a flawed approach:^28^ near-complete removal of these marginated pools would require draconian perfusion methods (eg, using EDTA or enzyme-containing perfusate).^20^ ^27^ Crucially, intravascular monocytes have also been observed in transplant lungs by histological examination.^13^ This is consistent with our novel EM data, which clearly confirm that monocytes are located in the pulmonary microvasculature, and indicate a likely interaction with the endothelium. The ability of marginated monocytes to withstand perfusion was attributed to inflammation within moribund donors, leading to upregulated expression of leukocyte adhesion molecules.^13^ Viable donor monocytes have also been found in the circulation of lung recipients in numbers that were disproportionately higher than other leukocyte subpopulations.^14^ In addition, the presence of significant numbers of blood monocytes had been confirmed in human lung tissue, using a combination of flow cytometry, cell sorting and histological analysis.^29^ Thus, although not an ‘anchored’ resident cell population such as alveolar macrophages, lung-marginated intravascular monocytes seem to be a bona fide passenger leukocyte population.

Despite their presence in mouse and human lungs, the potential contribution of lung-marginated monocytes to lung I/R injury has largely been unrecognised.^26^ In models of lung I/R, injury has been attributed to lung macrophages, with a delayed secondary role of vascular neutrophils.^30^ However, we and others have previously demonstrated that lung margination of monocytes can play a significant role in both direct and extra-pulmonary models of acute lung injury.^10–12^ ^31^ Moreover, unlike I/R in extra-pulmonary organs where anoxia and re-oxygenation can result in a diffuse injury, I/R injury during lung transplantation develops initially under normoxic conditions, with inflammation propagated locally by endothelial cell sensing of flow cessation.^32^ In spite of this localised ‘vascular’ origin of lung I/R injury, the role of marginated monocytes in direct contact with endothelial cells has been overlooked until now.

We found that monocytes present during I/R became activated, indicating their potential to enhance pulmonary vascular inflammation in mouse lungs. Using clodronate-liposome treatment, we observed reversal of the I/R-induced injury. When combined with an isolated organ-perfusion system, intravenous clodronate-liposome depletion provides an optimal method to evaluate the localised role of intravascular mononuclear phagocytic cells in isolation. Alternative monocyte depletion methods available were not suitable for this purpose: in the case of mAb anti-CCR2 treatment monocyte depletion is limited to the Ly6C^High^ subset, while monocyte-macrophage depletion in the CD11b diphtheria toxin transgenic mouse model is not compartment specific, potentially producing accumulation of apoptotic interstitial macrophages with resultant modulation of local inflammation.^21^ To further validate our clodronate-liposome-based findings, we performed monocyte transfer experiments in monocyte-depleted lungs and found a restoration of injury based on an increase in wet:dry ratios.

In human lung tissue, we identified monocytes as low granularity (side scatter)/CD14+/27E10^High^ cells, expressing high levels of HLA-DR and CCR2, mirroring the phenotype of blood monocytes. This combination of markers alone does not directly define their location, but their close resemblance to classical subset monocytes in blood, as well as our EM data and the recent more definitive identification of intravascular monocytes in healthy buffer-perfused human lungs by intravascular labelling and confocal microscopy,^33^ confirm that a large proportion of these cells should be intravascular. Lung monocyte numbers were found to correlate inversely with P:F ratios, suggesting that factors influencing passenger monocyte load, for example, pre-existing donor factors and/or events during retrieval, significantly contribute to post-transplant lung injury. Similarly, the relationships observed between monocyte CD86/TREM-1 expression and lung function/PGD scores, support a role for passenger monocytes. In our study, we did not observe a clear association between donor lung granulocyte parameters and lung dysfunction in recipients. As described before, IL-8 levels in donor lung tissue/BAL have been linked to PGD,^8^ ^30^ and there is convincing evidence in preclinical models for a critical role of infiltrating neutrophils in the development of I/R lung injury.^5^ ^6^ Our preliminary observations in human lungs, together with the IPL experiments, points to another important early role for the donor lung-marginated monocytes, that is, in addition to alveolar macrophages, they play a key role as orchestrators of lung I/R injury and PGD.

The mechanisms by which monocytes contribute to lung I/R were not defined here. Onset of normoxic lung ischaemia involves release of endothelial-derived reactive oxygen species (ROS),^34^ and proximity of marginated monocytes to endothelial cells could result in juxtacrine exposure to endothelial-derived ROS. ROS-mediated signalling can elicit similar responses in monocytes as those shown here, including TACE-mediated L-selectin shedding.^35^ The downstream effects of monocyte activation, including the reduction in KC and MIP-2 observed in monocyte-depleted lungs, and reduced neutrophil CD11b upregulation, suggest a central role for monocyte–neutrophil interactions in lung oedema development. Monocytes are key facilitators of neutrophil migration following intra-alveolar insults,^36^ and imaging-based studies suggest that intravascular monocytes are vital in orchestrating neutrophil behaviour during transplant-mediated I/R injury.^37^ ‘Patrolling’ Ly6C^Low^ monocytes that coordinate neutrophil interaction with injured endothelial cells in extra-pulmonary vascular beds,^38^ may also be applicable to the pulmonary vasculature.^39^

Our findings provide new evidence on how subclinical donor injury contributes to I/R injury and PGD. There are now substantial data that PGD is at least in part determined prior to implantation,^40^ and our results highlight a role for lung-marginated passenger monocytes in this process. Our concepts are also in keeping with a recent clinical study investigating lung monocyte/dendritic cell populations during the ex vivo lung perfusion.^9^ Although focused on the role of a different monocyte subset (non-classical monocytes) in organ rejection, they advocated the use of ex vivo lung perfusion as a potential mechanism of removing the passenger monocytes. With the emerging use of perfusion systems (eg, Organ Care System) for lung preservation and reconditioning,^41^ there is now an opportunity to remove/modify donor passenger leukocytes, in particular intravascular populations, before transplantation. Such ex vivo perfusion platforms may also allow advanced pharmacological or biological interventions to attenuate monocyte margination and/or activation to reduce PGD. This study provides important insights when considering such novel therapeutic possibilities.
