# Supplementary material for: Intravascular donor monocytes play a central role in lung transplant ischaemia-reperfusion injury
Source: Thorax. 2017 Apr 7;73(4):350–60. doi: 10.1136/thoraxjnl-2016-208977 (PMC5870457; doi:10.1136/thoraxjnl-2016-208977)
Supplement: Supplementary data [file thoraxjnl-2016-208977supp001.pdf]

SUPPLEMENT

SUPPLEMENTAL METHODS

Animal Protocols

All protocols were approved by the Imperial College Ethical Review Board and UK Home Office, in accordance with the Animals (Scientific Procedures) Act 1986, UK. Male C57BL/6 mice (Charles River Laboratories, Margate UK) aged 8–12 weeks (24–28g) were used for all protocols. Mice were housed in a specific pathogen free environment in individually ventilated cages, with no more than 5 mice per cage. They were provided with *ad libitum* access to food and water, environmental enrichment and a 12 h/12 h light-dark cycle. Animals were checked at least once daily by Named Animal Care and Welfare Officer. Additional monitoring and interventions were carried by Personal Licence (PIL) and/or Project Licence (PPL) holder, as specified in the PPL (70-7585).

Isolated perfused lung protocol

As previously described,[E1] mice received heparin (50 I.U.) via intravenous (i.v.) tail vein injection followed by intraperitoneal (i.p.) xylaxine (13 mg/kg) / ketamine (130 mg/kg) to induce general anaesthesia. The trachea was intubated via tracheostomy and connected to a custom-made mouse ventilator. Following two sustained inflation breaths (5 second, 25 cmH_2O), ventilation was commenced at a tidal volume of 6 ml/kg, at a rate of 80 breaths/minute, positive end expiratory pressure (PEEP) of 5 cmH_2O, with air. The mouse was subsequently exsanguinated via the inferior vena cava. The lower abdomen was removed to prevent contamination, and the sternum removed to provide access to the heart and great vessels. Ventilation was temporarily ceased and 5 cmH_2O CPAP remained for the duration of the thoracotomy with 5% CO_2/21% O_2 (to ensure normocapnia). A midline thoracotomy incision was performed, the sternum removed and the pulmonary artery cannulated and secured with a 3/0 silk suture. The left atrium was then cannulated though an incision in the apex, and secured with an occlusive 2/0 silk tie around the heart.

Perfusion for all IPL protocols was initially commenced via an open circuit (non-recirculating) configuration **(Lane 1, Figure E1)**, at a rate of 0.2 ml/min for the 1st minute, 0.4 ml/min for the 2nd minute and then 25 ml/kg/min thereafter. Effluent

perfusate was collected at point A **(Figure E1)** after discarding tubing (dead-space) perfusate. At the point of increasing the perfusion rate to 25 ml/kg/min, ventilation was recommenced with two sustained inflation breaths to 25 cmH_2O and then at a tidal volume of 6 ml/kg, 80/minute, PEEP 5 cmH_2O with 5% CO_2/21% O_2, as above.

All perfusion steps were performed with aseptically prepared phenol-free RPMI-1640 medium perfusate buffer supplemented with 0.1% sodium chloride, 0.08% sodium bicarbonate, glutamine (2 mM), and clinical grade human albumin solution (4%). To prevent bacterial growth during experiments, penicillin (100 U/ml) and streptomycin (0.1 mg/ml) were added to the perfusate buffer,[E2] and the circuit was cleaned with Mucasol, before and after runs with polymyxin B (both Sigma Aldrich, UK).

Lung compartment staining of leukocytes

Prior to administration of general anaesthesia, mice received 2 µg of anti-CD45 (Clone 30-F11, PE-CF594, BD Horizon, Oxford, UK) in 0.2 ml of saline i.v. via tail vein injection to label intravascular leukocytes. Following tracheostomy insertion, mice were exsanguinated and leukocytes in the alveolar compartment were labelled by intra-tracheal (i.t.) instillation of 2 µg of anti-CD45.2 (Clone 104, APC, BioLegend, London, UK) in 0.5 ml of saline and left *in situ* for 5 minutes. Lungs were then carefully excised, to avoid hilar lymphatics, and single cell suspensions for further antibody staining and flow cytometry, were produced as described below.

To assess retention of cells in the pulmonary vasculature during the IPL procedure, mice were injected i.v. with anti-CD45 antibody (PE-CF594) prior to anesthesia and mounting on the IPL. Open circuit (non-recirculating) perfusion and ventilation was then performed for 15 minutes (**Figure E2A**), followed then by i.t. anti-CD45.2 (APC) instillation and lung harvest. All effluent perfusate was collected (after discarding tubing dead-space volume).

Ischaemia/Reperfusion (I/R) protocol

To induce a 'no-flow' ischaemia, perfusion was halted after an initial 5 minute non-recirculating 'flush' period and ventilation switched to CPAP 5 cmH_2O with 5% CO_2/21% O_2, for 2 hours. Left atrial pressures were maintained at 2.5 mmHg by adjusting the height of the reservoir. At the end of the ischaemia period, the 5 minute non-recirculating flush step was repeated, and effluent perfusate collected. The perfusion system was then switched to its recirculating 'closed' configuration (**lane 2,**

Figure E1) and reperfusion performed at 40 ml/kg/min with ventilation recommenced (two sustained inflation breaths to 25 cmH_2O and then 6 ml/kg tidal volume, 80/minute, PEEP 5 cmH_2O with 5% CO_2/21% O_2), for 2 hours. At the end of the reperfusion period, lung and circuit perfusate was collected from a final 5 minute flush period (**Figure E2B**). There were no adverse events recorded in the control or test groups.

Monocyte depletion and adoptive transfer

For depletion of intravascular monocytes, mice were injected i.v. with 0.2 ml of liposomal clodronate (FormuMax Scientific, Palo Alto, CA, USA), 24 hours prior to IPL protocols, as previously described.[E1] No significant change in behaviour or weight from baseline was noted in this group of animals.

For adoptive transfer, circulating blood monocytes were isolated via an immune-magnetic bead negative selection mouse monocyte enrichment kit according to the manufacturer's instructions (Stemcell, Grenoble, France). Whole blood (~7 ml) was obtained from ~7 mice, after terminal anaesthesia (xylaxine/ketamine), by direct cardiac puncture using heparinised syringes. Red blood cells were lysed by ammonium chloride (1:9) treatment on ice and centrifuged at 300 $\times$ *g* at room temperature for 6 minutes. Cells were washed with EasySep™ buffer (Stemcell, Grenoble, France), at 300 $\times$ *g* for 10 minutes and resuspended in the same buffer supplemented with 5% rat serum. Cells were incubated in EasySep™ monocyte enrichment cocktail, (15 minutes at 4°C) before addition of the magnetic particles for a further 10 minutes at 4°C. Isolated monocytes were resuspended in the perfusate buffer and assessed for purity by via flow cytometry.

Isolated lungs from clodronate-liposome treated mice were prepared for monocyte transfer by an initial 5 minute pulmonary flush. Monocytes suspensions were then injected slowly into the pulmonary circulation (**via point B, Figure E1**). To equilibrate infused cells, recirculating perfusion (**lane 2, Figure E1**) was performed for 10 minutes prior to the standard I/R protocol described above (**Figure E2C**).

Quantification of lung oedema

On completion of IPL protocols, the left lungs were tied off with a 2/0 silk (for wet:dry analysis). Saline (0.9%, 0.5 ml) was then gently instilled via the tracheal tube into the remaining lobes, and lavaged a total of three times before fluid was aspirated. BAL samples were centrifuged at 400 $\times$ *g* for 7 minutes at 4°C and supernatants were removed, vortexed and stored at -80°C. To quantify protein content, BAL samples

were analysed in triplicate via the Bradford method[E3] utilising Coomassie Brilliant Blue G-250 dye (BioRad, Hemel Hempstead, UK), human albumin solution (Baxter, Newbury, UK) standards, and analysed at 595nm absorbance, with Revelation software v4.22 200 on an MRX II absorbance plate reader (Dynex Technologies, Worthing, UK). For wet:dry weight ratio analysis, the left lungs were removed, blotted and weighed before being placed in a 60°C oven. Lungs were weighed daily until no further weight loss was observed and wet:dry weight ratios calculated.

Preparation of single cell suspension from mouse lungs

For preparation of single cell suspensions, the right lungs lobes were harvested following BAL, blotted and weighed, before undergoing mechanical disruption with a gentleMACS™ Dissociator (Miltenyi Biotec Ltd, Bisley, UK) with 2 ml intracellular fixative (eBioscience, Hatfield, UK) in MACS C tubes (Miltenyi Biotec), for 1 minute. The fixation reaction was stopped by the immediate addition of 8 ml of ice-cold flow cytometry wash buffer (PBS with 2% FCS, 0.1% sodium azide, and 2 mM EDTA; all from Sigma Aldrich, Gillingham, UK). This technique allows rapid dissociation of tissue and fixation of cells, minimising staining by residual antibodies (intravascular or intra-alveolar) and preventing enzymatic degradation of epitopes.[E4, E5] For quantification of L-selectin expression, lung cells suspensions were prepared without fixative, due to the loss of antibody (clone MEL-14) reactivity on fixed cells. Right lung lobes were disrupted in M dissociation tubes (Miltenyi Biotec) in ice-cold flow buffer, supplemented with 10 µM Batimastat (BB) 94 (Vernalis, Winnersh, UK), a hydroxamate-based metalloproteinase inhibitor, to prevent L-selectin cleavage.

Lung cell suspensions were sieved through a 40 µm cell strainer (BD Biosciences, Oxford, UK) with gentle disaggregation of any tissue remnants using a syringe plunger and centrifuged at 400 × *g* for 5 minutes at 4°C. Cells were washed again by centrifugation in 4 ml of flow buffer, and then resuspended to 1 ml, prior to antibody staining for flow cytometry.

Flow cytometry of mouse cells

Lung cell suspensions and perfusate samples were incubated with the antibody combinations listed in **Supplemental Table E1** for 30 minutes in the dark at 4°C. For L-selectin quantification on unfixed cells, flow buffer supplemented with 10 µM BB-94 was used for staining and washing samples. Samples were washed once and resuspended in 300 µl of flow buffer and acquired using a Cyan flow cytometer

(Beckman Coulter, High Wycombe, UK). AccuCheck counting beads (ThermoFisher, Loughborough, UK) were added to samples to determine cell counts. Cell counts were adjusted per total lungs, based on weight calculation. Data were analysed with FlowJo (V.10.0.8, Ashland, OR, USA)

Table E1

Anti-mouse antibodies

Fluorescence-conjugated anti-mouse antibodies utilised in the dual-compartment staining and final lung leukocyte enumeration and activation quantification experiments.

* ***FITC**: fluorescein, isothiocyanate, **PE**: phycoerythrin, **PerCP**: perinidin chlorophyll protein, **APC**: allophycocyanin, **Cy**: cyanine.*

**Channel:**	**Dual compartment cocktail**<br>Antibody (clone, company):	**Final cocktail**<br>Antibody (clone, company):
**FITC/488**	F4/80<br>(BM8, eBioscience, Hatfield, UK)	F4/80<br>(BM8, eBioscience, Hatfield, UK)
**PE**	CD11b<br>(M1/70, BioLegend, London, UK)	L-selectin<br>(MEL-14, BD Biosciences, Oxford, UK)<br>CD86<br>(GL1, eBioscience, Hatfield, UK)
**PE-CF 594**	Intravenous CD45<br>(30-F11, BD Biosciences, Oxford, UK)	CD11b<br>(M1/70, BD Horizon, Oxford, UK)
**PerCP/PECy5**	MHC II<br>(M5/114.15.2, BioLegend, London, UK)	MHC II<br>(M5/114.15.2, BioLegend, London, UK)
**PECy7**	Ly6C<br>(HK 1.4, BioLegend, London, UK)	Ly6C<br>(HK 1.4, BioLegend, London, UK)
**APC**	Intra-tracheal CD45.2<br>(104, BioLegend, London, UK)	Ly6G<br>(1A8, BioLegend, London, UK)
**APC-Cy7/eFluor 780**	CD11c (N418, eBioscience, Hatfield, UK)	CD11c<br>(N418, eBioscience, Hatfield, UK)

Measurement of inflammatory markers

Quantification of all mouse chemokines and cytokines (MCP-1, KC, sRAGE, MCP-1, IL-6, IL-1β and TNFα) in lung perfusates and BAL was performed using the DUOSET® sandwich ELISA system (R&D Systems, Abingdon, UK) according to the manufacturer's instructions. Plates were read at 450 nm (wavelength correction 540nm) on the MRX II absorbance plate reader and analysed with Revelation software v4.22 200.

Collection of human lung tissue

Human lung samples were harvested and stored in accordance with the POPSTAR ethics and study protocol 13/LO/0152 (Royal Brompton & Harefield NHS Foundation Trust). The principle objective of this prospective cohort study was to comprehensively evaluate surgical, perioperative and technological aspects of lung transplantation, combined with establishment of a biobank and a mechanistic study towards inflammatory and metabolic components of lung injury. A biopsy was taken from the donor lung right lower lobe with a Covidien GIA stapler 80 mm (Medtronic, Watford, UK). Each biopsy measured approximately 3 cm x 1 cm x 1 cm. Human healthy volunteer blood was sampled from the staff of Imperial College London.

A total of 13 lung samples were obtained from 11 'donation after brainstem death' (DBD), and 2 'donation after circulatory death (DCD) donors'. **Supplemental Table E2** summarises demographic, physiological and outcome data. P:F ratios were calculated from the poorest daily partial pressure of arterial oxygen (PaO_2; mmHg) and the corresponding fraction of inspired oxygen. Primary graft dysfunction was defined as Grade 0 in the absence of radiographic infiltrates suggestive of pulmonary oedema. Grade 1 PGD was defined as: P:F ratio >300mmHg with presence of allograft infiltrates on chest x-ray, Grade 2: P:F ratio 200-300mmHg and Grade 3: P:F ratio <200mmHg, both with allograft chest x-ray changes, at 24, 48 and 72 hours.

Table E2

Transplant demographics, physiological variables and outcomes.

PF- PaO_2:FiO_2 ratio; PGD- Primary Graft Dysfunction; ICU – Intensive Care Unit

***Transplant Demographics, Physiological Variables and Outcomes* (*n*= 13)**	
**Donor age [years],** median (IQR)	44 (30-60)

**Male donor sex,** n (%)	6 (46)
**Intracranial haemorrhage,** n (%)	7 (54)
**Donor PF ratio [mmHg],** median (IQR)	456 (347-521)
**Donor score,** median (IQR)	4 (2-7)
**Ischaemic time [mins],** median (IQR)	299 (265-468)
**Recipient age [years],** median (IQR)	55 (46-62)
**Male recipient sex,** n (%)	5 (38)
**Recipient PF ratio at 24 hours [mmHg],** mean ± SD	247±101
**Recipient PF ratio at 48 hours [mmHg],** mean ± SD	322±94
**Recipient PF ratio at 72 hours [mmHg],** mean ± SD	354±86
**PGD 2-3 at 24 hours,** n (%)	6 (46)
**PGD 2-3 at 48 hours,** n (%)	4 (31)
**PGD 2-3 at 72 hours,** n (%)	3 (23)
**Mechanical ventilation [hours],** median (IQR)	53 (26-308)
**ICU length stay [days],** median (IQR)	6 (4-21)
**Hospital mortality,** n (%)	2 (15)

Donor lungs underwent pulmonary flushing with Perfadex (Vitrolife, Göteborg, Sweden); DBD lungs: 4 L anterograde/1 L retrograde perfusion; DCD lungs: 3 L antegrade/2 L retrograde perfusion.[E6] Pulmonary perfusion pressures were maintained between 10-15 mmHg during pulmonary flushing and lungs transported to the hospital inflated (FiO_2 0.5) on ice. Lung biopsies were taken thereafter, immediately prior to implantation, and stored and transported from the hospital to the laboratory for analysis in a humidified container on ice.

Preparation of single cell suspensions from human lung tissue

Lung tissue was subdivided into two pieces and weighed. One portion was used to determine dry weight (as described for mouse tissue above) and the other was weighed and minced manually with a scalpel blade before incubating with Liberase (100 µg/ml Roche, Burgess Hill, UK) and DNAse (62.5 µg/ml, Sigma Aldrich, Gillingham, UK) at 37°C for 20 minutes with continuous mixing. Tissue suspensions

were then suspended in 5ml of flow buffer and sieved through a 40µm cell strainer, with gentle disaggregation of remaining tissue using a syringe plunger, and centrifuged 7 minutes at 4°C at 300 x *g*. Two additional washes were performed prior to suspension of cells in flow buffer for antibody staining.

Flow cytometry of human lung and blood.

Lung cell suspensions and heparinised blood from healthy volunteers (as a reference sample) were incubated in the dark for 30 minutes at 4°C with the antibodies listed in **Supplemental Table E3**, and washed in flow buffer for 7 minutes at 4°C at 300 × *g*. To identify monocytes and granulocytes in human lung cell suspensions, we used the monoclonal antibody 27E10,[E7] which is specific for the intracellular S100A8/A9 heterocomplex (known also as MRP8/MRP14 or calprotectin). S100A8/A9 is highly expressed in monocytes and neutrophils, and reactivity with the 27E10 antibody has previously been used in combination with other cell markers to distinguish invasive monocytes from resident alveolar macrophages in BAL fluid.[E8, E9] Surface marker pre-stained cells were fixed and permeabilised by addition of 0.5 ml of Cytofix/Cytoperm™ (BD Biosciences, Oxford, UK) and incubated for 5 minutes at room temperature. After two washes with permeablisation buffer (PBS, 2% FCS, 0.2% saponin, 0.1% sodium azide), cell suspensions were incubated with FITC-conjugated-27E10 antibody in the dark for 15 minutes at 4°C. They were washed and resuspended with 10 µl of counting beads prior to acquisition on the Cyan flow cytometer. Counting beads were added to samples to determine cell counts, which were adjusted to cells/ per gram dry lung weight.

Table E3

Anti-human antibodies

Fluorescence-conjugated anti-human antibodies utilised for lung and blood leukocyte identification, enumeration and quantification of activation.

Fluorophore:	***Antibody (clone, company):***
**FITC/488**	S100A8/A9 (27E10*, Santa Cruz, Dallas, TX, USA)
**PE**	CD66B (G10FS, BioLegend, London, UK)
**PE Texas Red/CF 594**	CD11b (M1/70, BD Horizon, Oxford, UK)
**PerCP/PECy5**	CD14 (M5E2, BioLegend, London, UK)

**PECY7**	CD16 (3G8, BioLegend, London, UK)
**APC/Ax**	CCR2 (48607, BD Biosciences, Oxford, UK)<br>HLA-DR (L342, BioLegend, London, UK)<br>TREM-1 (TREM-26, BioLegend, London, UK)<br>CD86 (IT2.2, BioLegend, London, UK)
**APC-Cy7/eFluor 780**	CD45 (HI30, BioLegend, London, UK)

** 27E10 was stained for at a later stage following cell fixation/permeabilisation.*

Histology and Immunohistochemistry

5μm paraffin sections of lung tissue were dewaxed and rehydrated to water and incubated in Mayer's Haematoxylin, washed and stained with 1% Eosin before dehydration and mounting in DPX (Distyrene/plasticiser/xylene, all from VWR International Ltd., Lutterworth, UK).

For immune-localisation, after heat-induced antigen retrieval, sections were blocked for endogenous peroxidases with 0.3% hydrogen peroxide, followed by blocking with 3% bovine serum albumin. Sections were then incubated with primary antibody mouse monoclonal anti-CD45 (Abcam PLC, Cambridge, UK), washed and incubated with a second layer of Biotinylated goat anti-rabbit immunoglobulins (GAM IgG, Vector Laboratories Ltd., Peterborough, UK). After washing, sections were incubated with Avidin-Biotin Complex ABC (Vector Laboratories Ltd.). Reactivity was detected using diaminobenzidine tetrahydrochloride (Sigma Aldrich Co Ltd., Irvine, UK) 25mg/ml and hydrogen peroxide (0.01%). Sections were then counter stained with haematoxylin and viewed on Zeiss Axioskop microscope (Carl Zeiss Ltd., Cambridge, UK. Digital micrographs were taken using Nikon DMX1200 camera (Nikon UK Ltd., Kingston, UK).

Electron micrograph preparation

Biopsy was taken using a GIA staple (Covidien, Medtronic) from the donor lungs prior to implantation into the recipient. After primary fixation in 2.5 % glutaraldehyde in 0.1 M sodium cacodylate buffer (Agar Scientific Ltd., Essex, UK) followed by secondary fixation with 1% osmium tetroxide in 0.1 M sodium cacodylate buffer (Agar Scientific Ltd., Essex, UK) for one hour at room temperature, lung tissue was dehydrated through ascending concentrations of acetone to 100%, followed by 30 minutes in propylene oxide (Agar Scientific Ltd., Essex, UK), prior to infiltration with 1:1 propylene oxide/ araldite CY212 resin overnight in the specimen rotator and in araldite CY212 resin for 4 hours and polymerization for 18 hours at 60°C. Ultra-thin sections (100 nm)

obtained using a Diatome diamond knife, floated onto distilled water, collected on grids and stained with 2% uranyl acetate and lead citrate for 10 minutes in each solution. Electron micrographs were acquired by Transmission Electronic Microscope (TEM) JEOL 1200 EX, and GatanDigitalMicrograph.

Statistics

Based on our previous experience of *in* and *ex vivo* studies in this area, we estimate that the effect size to be detected would be roughly 1.5-2.5. Thus, a statistical power of 0.80 is achieved at n=8 with an effect size of 1.5 and n=4 with an effect size of 2.5, assuming normal distribution, equal variances and two-tail tests. This implies that an average of 5-6 repeats are required per group.

Statistical analyses were performed using SPSS version 22 (IBM, Portmouth, UK) and Graphpad Prism version 6 (Graphpad, La Jolla, CA, USA). Data normality was determined using QQ plots and Shapiro-Wilk tests. Group comparisons were made by Student's t-tests (parametric) or Mann-Whitney U tests (non-parametric), or by ANOVA with Bonferroni tests (parametric) or Kruskal-Wallis with Dunn's tests (non-parametric) for more than two groups. Correlation analysis was performed using the Spearman rank tests. Data are presented as mean ± SD (parametric) or median with interquartile range (non-parametric). A two-sided p value less than 0.05 was considered statistically significant.

SUPPLEMENTAL FIGURE LEGENDS

Figure E1

The isolated perfused lung (IPL) circuit.

The IPL consists of a peristaltic roller pump with 2 lanes. Lane 1 infused fresh perfusate into the lungs, which was collected at point A; lane 2 allowed perfusate to be re-circulated around the circuit. Perfusate was warmed by passing it through a heat exchanger of coiled tubing, within the lung chamber (at 37°C). Left atrial pressure was maintained at 2.5mmHg by adjusting the height of the reservoir.

Figure E2

IPL protocols

Lung washout for compartment analysis (**Protocol A**), consisted of 15 minutes of non-recirculating perfusion via lane 1. Perfusate was collected at point A.

The I/R protocol consisted of a 5 minute initial 'flush' with non-recirculating perfusion, which was collected at point A. Perfusion and ventilation were then halted for 2 hours of 'stop-flow' ischaemia. A further 5 minute flush period was then completed and the lungs were reperfused for a further 2 hours (non-recirculating) before a final 5 minute flush (**Protocol B**).

For adoptive transfer experiments, monocytes were infused into the pulmonary circulation via point B, following the initial 5 minute flush. They were re-circulated for 10 minutes prior to the completion of the standard I/R protocol (**Protocol C**).

Figure E3

Human Lung Histology and Immunohistochemistry

A-B: Micrographs of paraffin-embedded lung sections, obtained from donor lungs at the end of the cold ischaemia period, (following pulmonary flushing and immediately prior to implantation in the recipient), demonstrate the presence of leucocytes in the lung, including tri- and bi-lobe nucleated neutrophils, and monocytes with their typical horseshoe-like nuclei. Sections were stained for haematoxylin and eosin. Scale = 50 μm as shown.

C: Micrographs of frozen lung sections obtained from donor lungs at the end of the cold ischaemia period (following pulmonary flushing and prior to implantation into the recipients). Sections were stained for CD45, a pan-leucocyte marker. CD45-positive

cells (in brown) appear in the context of the alveolar wall. Scale = 50 μm as shown.

These images, while suggesting significant intravascular margination of leukocytes, do not allow precise identification of compartmental (intra-alveolar, interstitial or intravascular) location of these cells within the complex, but very thin, alveolar wall structure. Hence, we undertook flow cytometry with *in vivo* dual-labelling compartmental analysis for mouse lungs, and flow cytometry combined with electron microscopy for human samples.

REFERENCES

E1. Wakabayashi K, Wilson MR, Tatham KC, et al. Volutrauma, but not atelectrauma, induces systemic cytokine production by lung-marginated monocytes. *Crit Care Med* 2014;42(1):e49-57

E2. Salzer WL, McCall CE. Primed stimulation of isolated perfused rabbit lung by endotoxin and platelet activating factor induces enhanced production of thromboxane and lung injury. *J Clin Invest* 1990;85(4):1135-43

E3. Bradford MM. A rapid and sensitive method for the quantitation of microgram quantities of protein utilizing the principle of protein-dye binding. *Anal Biochem* 1976;72:248-54

E4. Patel BV, Tatham KC, Wilson MR, et al. In vivo compartmental analysis of leukocytes in mouse lungs. *Am J Physiol Lung Cell Mol Physiol* 2015;309(7):L639-52

E5. O'Dea KP, Dokpesi JO, Tatham KC, et al. Regulation of monocyte subset proinflammatory responses within the lung microvasculature by the p38 MAPK/MK2 pathway. *Am J Physiol Lung Cell Mol Physiol* 2011;301(5):L812-21

E6. Andrews PA, Burnapp L, Manas D, et al. Summary of the British Transplantation Society guidelines for transplantation from donors after deceased circulatory death. *Transplantation* 2014;97(3):265-70

E7. Zwadlo G, Schlegel R, Sorg C. A Monoclonal-Antibody to a Subset of Human-Monocytes Found Only in the Peripheral-Blood and Inflammatory Tissues. *Journal of Immunology* 1986;137(2):512-18

E8. Striz I, Pokorna-Sochurkova H, Zheng L, et al. Calprotectin expression and mononuclear phagocyte subpopulations in peripheral blood and bronchoalveolar lavage. *Sarcoidosis Vasculitis and Diffuse Lung Diseases* 2001;18(1):57-63

E9. Rosseau S, Hammerl P, Maus U, et al. Phenotypic characterization of alveolar monocyte recruitment in acute respiratory distress syndrome. *Am J Physiol Lung Cell Mol Physiol* 2000;279(1):L25-35

Figure E1 The isolated perfused lung (IPL) circuit

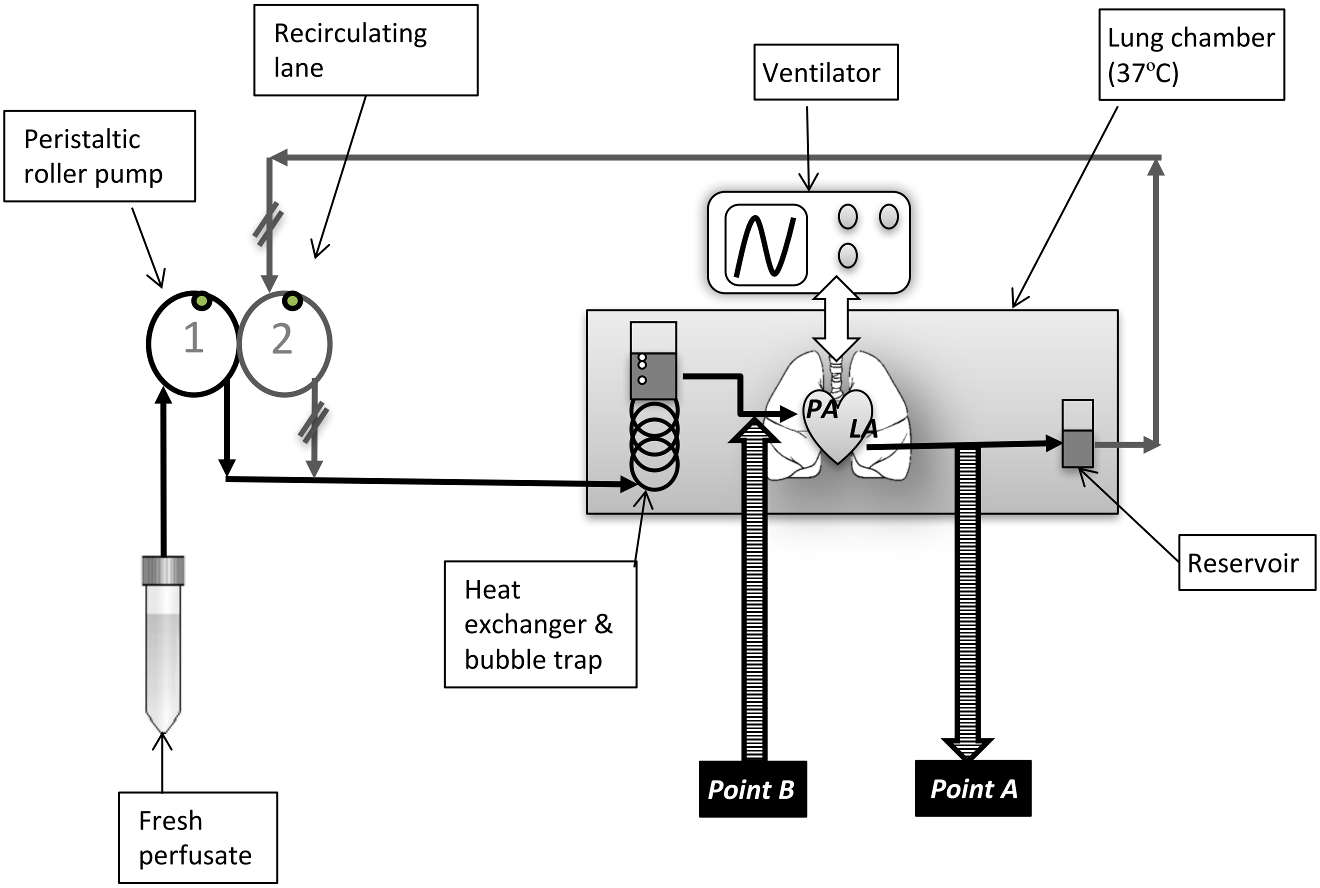

The IPL consists of a peristaltic roller pump with 2 lanes. Lane 1 infused fresh perfusate into the lungs, which was collected at point A; lane 2 allowed perfusate to be re-circulated around the circuit. Perfusate was warmed by passing it through heated exchanger of coiled tubing, within the lung chamber (at 37°C). Left atrial pressure was maintained at 2.5mmHg by adjusting the height of the reservoir.

Figure E2 Isolated perfused lung protocols

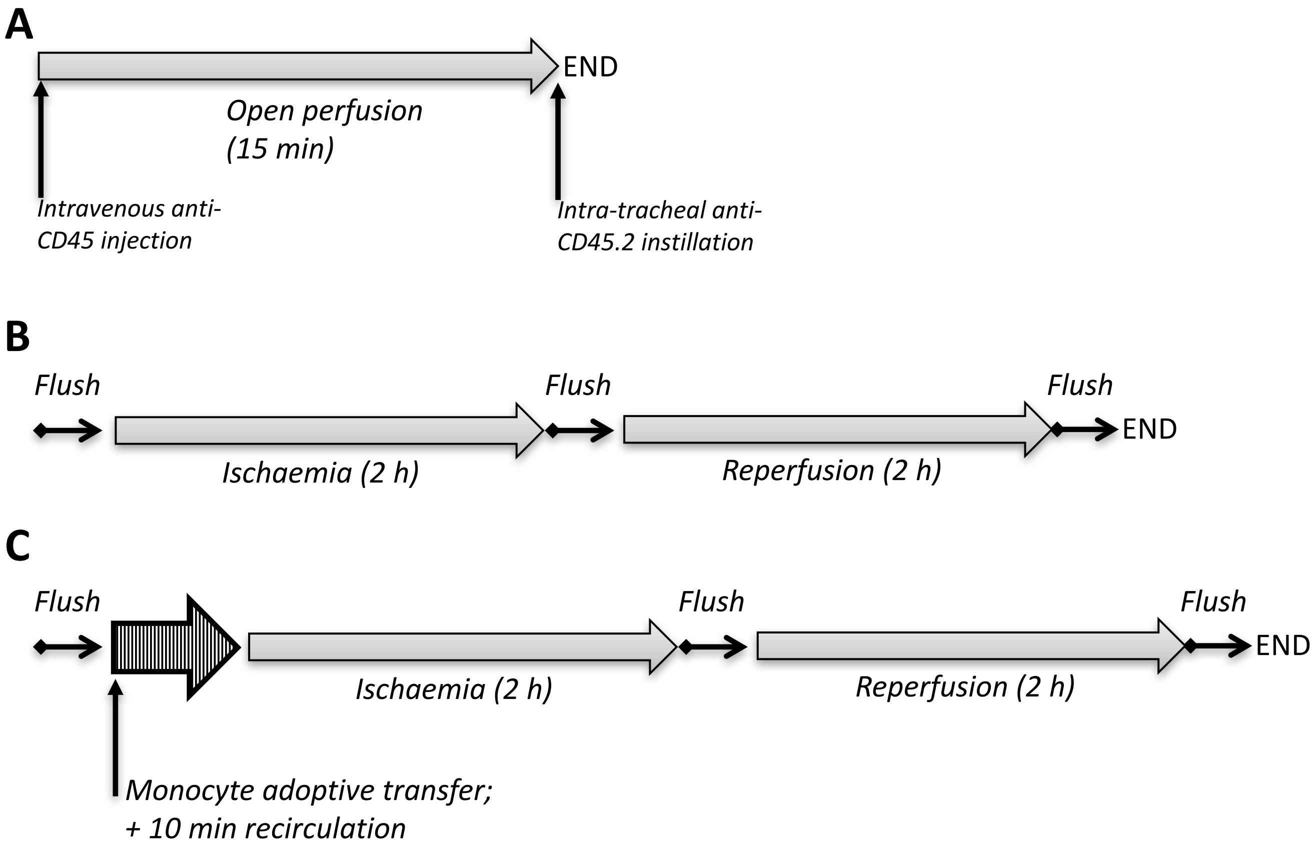

Lung washout for compartment analysis (protocol A), consisted of 15 minutes of non-recirculating perfusion via lane 1. Perfusate was collected at point A.

The I/R protocol consisted of a 5 minute initial 'flush' with non-recirculating perfusion, which was collected at point A. Perfusion and ventilation were then halted for 2 hours of 'stop-flow' ischaemia. A further 5 minute flush period was then completed and the lungs were reperfused for a further 2 hours (non-recirculating) before a final 5 minute flush (Protocol B).

For adoptive transfer experiments, monocytes were infused into the pulmonary circulation via point B, following the initial 5 minute flush. They were re-circulated for 10 minutes prior to the completion of the standard I/R protocol (Protocol C).

Figure E3 Lung Histology and Immunohistochemistry

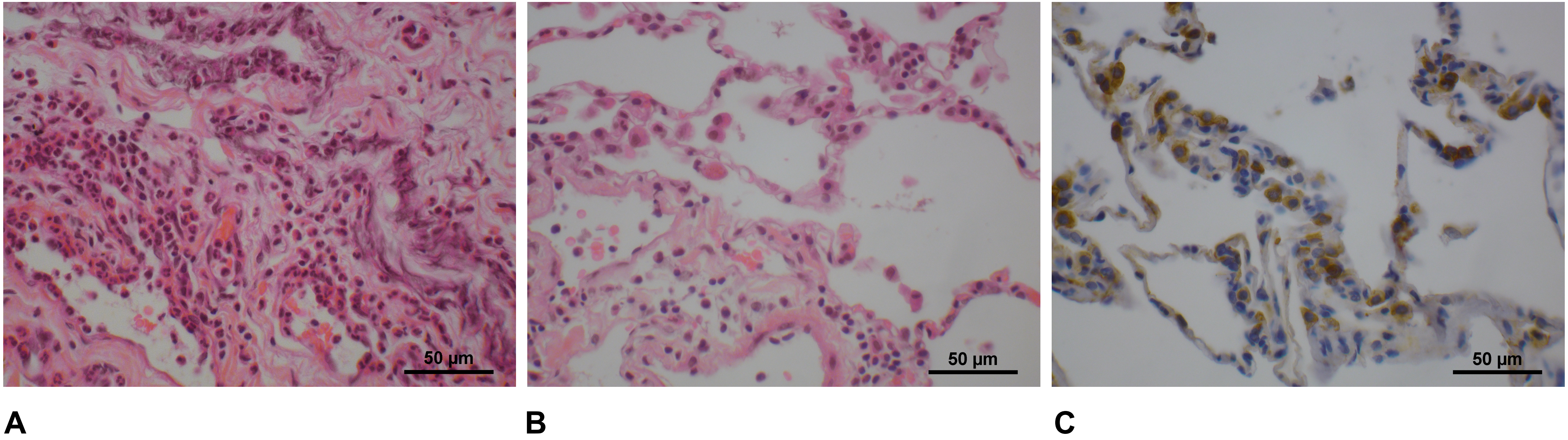

A-B Micrographs of paraffin-embedded lung sections, obtained from donor lungs at the end of the cold ischaemia period, (following pulmonary flushing and immediately prior to implantation in the recipient), demonstrate the presence of leucocytes in the lung, including tri- and bi-lobe nucleated neutrophils and monocytes with their typical horseshoe-like nuclei. Sections were stained for haematoxylin and eosin.
Scale = 50 μm as shown.

C Micrographs of frozen lung sections obtained from donor lungs at the end of the cold ischaemia period (following pulmonary flushing and prior to implantation into the recipients). Sections were stained for CD45, a pan-leucocyte marker. CD45-positive cells (in brown) appear in the context of the alveolar wall.
Scale = 50 μm as shown.

These images, while suggesting significant intravascular margination of leukocytes, do not allow precise identification of compartmental (intra-alveolar, interstitial or intravascular) location of these cells within the complex, but very thin, alveolar wall structure. Hence, we undertook flow cytometry with *in vivo* dual-labelling compartmental analysis for mouse lungs, and flow cytometry combined with electron microscopy for human samples.
